# Time course of arterial remodelling in diameter and wall thickness above and below the lesion after a spinal cord injury

**DOI:** 10.1007/s00421-012-2400-2

**Published:** 2012-04-17

**Authors:** Dick H. J. Thijssen, Patricia C. E. De Groot, Arne van den Bogerd, Matthijs Veltmeijer, N. Timothy Cable, Daniel J. Green, Maria T. E. Hopman

**Affiliations:** 1 Department of Physiology, Radboud University Nijmegen Medical Centre, Philips van Leydenlaan 15, 6525 EX Nijmegen, The Netherlands; 2 Research Institute for Sport and Exercise Science, Liverpool John Moores University, Liverpool, UK; 3 School of Sport Science, Exercise and Health, The University of Western Australia, Crawley, Australia

**Keywords:** Intima-media thickness, Arterial wall, Cardiovascular risk, Physical inactivity

## Abstract

Physical inactivity in response to a spinal cord injury (SCI) represents a potent stimulus for conduit artery remodelling. Changes in conduit artery characteristics may be induced by the local effects of denervation (and consequent extreme inactivity below the level of the lesion), and also by systemic adaptations due to whole body inactivity. Therefore, we assessed the time course of carotid (i.e. above lesion) and common femoral artery (i.e. below lesion) lumen diameter and wall thickness across the first 24 weeks after an SCI. Eight male subjects (mean age 35 ± 14 years) with a traumatic motor complete spinal cord lesion between T5 and L1 (i.e. paraplegia) were included. Four subjects were measured across the first 6 weeks after SCI, whilst another four subjects were measured from 8 until 24 weeks after SCI. Ultrasound was used to examine the diameter and wall thickness from the carotid and common femoral arteries. Carotid artery diameter did not change across 24 weeks, whilst femoral artery diameter stabilised after the rapid initial decrease during the first 3 weeks after the SCI. Carotid and femoral artery wall thickness showed no change during the first few weeks, but increased both between 6 and 24 weeks (*P* < 0.05). In conclusion, SCI leads to a rapid and localised decrease in conduit artery diameter which is isolated to the denervated and paralyzed region, whilst wall thickness gradually increases both above and below the lesion. This distinct time course of change in conduit arterial diameter and wall thickness suggests that distinct mechanisms may contribute to these adaptations.

## Introduction

Physical inactivity is a potent stimulus for vascular remodelling of conduit arteries (Thijssen et al. 2010), which may contribute to the associated increased cardiovascular risk (Green et al. 2008). Spinal cord injury (SCI) results in marked vascular remodelling below the level of the lesion (Thijssen et al. 2011c), which is largely accomplished within 3 weeks after the injury and relates to the extreme deconditioning induced by denervation (de Groot et al. 2006). In contrast, conduit arteries diameter above the lesion, which may reflect changes in whole body physical activity levels, have previously been reported in cross-sectional comparisons to be similar to that observed in matched able-bodied controls (de Groot et al. 2004). Although arterial diameter and wall thickness are related (Thijssen et al. 2011d), very little is known about the impact of SCI on conduit artery wall thickness above and below the lesion. Also, few studies have followed the time course of adaptation in artery characteristics following SCI within affected subjects and none, to our knowledge, have examined (time course of) arterial responses above and below the lesion, an approach which may provide insight into localised effect of denervation versus systemic effects of physical inactivity.

The aims of this study were: (1) to examine whether SCI leads to adaptations in wall thickness due to whole body physical inactivity (i.e. above lesion) or local denervation (i.e. below lesion) and (2) to examine the time course of changes in arterial wall thickness across a period of physical inactivity. To this end, we repeatedly assessed diameter and wall thickness of the femoral and carotid arteries across the first 24 weeks after an SCI. We hypothesised the presence of larger changes in femoral artery wall thickness (i.e. below lesion) than that observed in the carotid artery (i.e. above lesion). Also, we expect a similar time course in the carotid and femoral artery after an SCI.

## Methods

### Subjects

Eight male SCI individuals with a traumatic motor complete spinal cord lesion [American Spinal Injury Association (ASIA) Impairment Scale (AIS) grade A or B] between T5 and L1 (i.e. paraplegia) participated in this longitudinal study. Patients were included as soon as their clinical and personal situations allowed conversation and measurements needed for this study. All subjects underwent a regular medical examination in the rehabilitation clinic. Based on patient records, we excluded subjects with a known history of cardiovascular disease, diabetes mellitus type I or II, hypercholesterolemia (≥6.5 mmol/L or lipid-lowering drugs) or high blood pressure (≥160 or ≥90 mmHg systolic or diastolic blood pressure, or anti-hypertensive drugs) from the study. Individual subject characteristics are presented in Table 1. We included two subjects that smoked until the time of accident. The Ethical Committee of the Radboud University Nijmegen Medical Centre approved the study and all subjects provided written, informed consent before participating.

**Table 1:** Subject characteristics for all participants in our study

Subject	Age (years)	Height (cm)	Weight (kg)	SBP (mmHg)	DBP (mmHg)	Lesion level (AIS grade)
1	36	180	75	119	72	T10 (A)
2	28	174	55	105	69	T9 (A)
3	65	180	71	138	68	L1 (A)
4	33	165	60	119	81	T12 (B)
5	36	175	60	108	68	T5 (A)
6	21	168	50	115	60	T12 (A)
7	43	175	65	120	70	T12 (B)
8	20	183	58	120	70	L1 (B)
Mean	35 ± 14	175 ± 6	62 ± 8	118 ± 10	70 ± 6	

Subjects 1–4 were tested during weeks 2–6, and subjects 5–8 were tested between 6 and 24 weeks. The AIS grade refers to the ASIA (American Spinal Injury Association) Impairment Scale, in which ‘A’ represents a complete motor and sensor lesion and ‘B’ relates to an incomplete lesion with (partly) preserved sensory, but not motor function

*SBP* systolic blood pressure, *DBP* diastolic blood pressure, *AIS* American spinal injury, *T* lesion at thoracic level, *L* lesion at lumbar level)

### Experimental design

Based on the severity of the trauma-related complications, we measured four subjects repeatedly from weeks 3 to 6 post-injury. Another four patients were repeatedly measured from week 8 until week 24 post-injury. All measurements were performed in resting supine position under standardised conditions after an overnight fast at the University Nijmegen Medical Centre or at the rehabilitation clinic. Subjects were asked to empty their bladder before examination and were asked to abstain from alcohol, caffeine and nicotine for at least 12 h before the measurements. The same investigator performed all measurements.

### Measurements

Resting red blood cell velocity and diameter of the common femoral artery and carotid artery were obtained by using echo Doppler ultrasound with a 5- to 7.5-MHz broadband linear array transducer. For the carotid artery, images were made 1.5 cm proximal from the bifurcation of the left common carotid artery. The images of the common femoral arteries were obtained just below the inguinal ligament, about 2 cm proximal of the bifurcation into the deep and superficial femoral artery. For the measurements, two consecutive images in the longitudinal view were frozen at the peak-systolic and end-diastolic phase. All images of the femoral and left carotid artery were stored on videotape. Finally, the video files were converted to DICOM (Digital Imaging and Communications in Medicine) files for further analysis.

For assessment of red blood cell velocity, the sample volume was adjusted to cover the width of the vessel and, thus, the complete blood velocity distribution. The angle of inclination for the velocity measurements was consistently below 60°, and the vessel area was adjusted parallel to the transducer (Thijssen et al. 2011a). From each artery, 4 images with a total of 10–12 velocity profiles were obtained and traced manually afterwards by a single investigator (de Groot et al. 2006). A part of this data has been published previously in a study focusing on adaptations of arterial diameter after an SCI (de Groot et al. 2006). These data are included here to establish the effects of physical inactivity on arterial diameter.

### Data analysis

#### Arterial wall thickness

Wall thickness and diameter analysis of the arteries were performed using a custom-designed edge-detection and wall-tracking software. This DICOM-based software is largely independent of investigator bias and has been previously described in detail (Potter et al. 2007, 2008). Briefly, the initial video signal was encoded and stored as a digital file, and converted to a DICOM file after the completion of the test. Software analysis was performed at 30 Hz using an icon-based graphical programming language and toolkit (LabView 6.02, National Instruments, Austin, TX). By identifying a region of interest (ROI) on each first frame of every individual study, capturing both walls of the artery, an automated calibration was made of diameters on the B-mode image. Within the identified ROI in the diameter image, a pixel-density algorithm automatically identified the angle-corrected near and far wall e-lines for every pixel column for diameter assessment. The same algorithm was used to identify the far wall media-adventitia interface. Detection of the near and far wall lumen edges and the far wall media-adventitia interface was performed on every frame selected. This technique has a good inter-observer reproducibility, with a coefficient of variation of 5.1 % (Potter et al. 2007). The mean diameter and wall thickness were then calculated by using the formula: 1/3 × systolic diameter or wall thickness + 2/3 × diastolic diameter or wall thickness. The observer was blinded when the file was recorded. Subsequently, all files were checked by at least one independent researcher who was also blinded when the file was recorded.

#### Conduit artery blood flow

The average of 10–12 Doppler spectra waveforms was used to calculate mean red blood cell velocity (*V*_mean_). From each velocity profile, the flow velocity integral was manually traced. Mean blood flow in millilitre per minute was calculated using mean resting diameter (as calculated above) according to the recent guidelines (Thijssen et al. 2011a).

### Statistical analysis

Statistical analyses were performed using SPSS 17.0 computer software (SPSS, Chicago, Illinois). To asses a time trend, time was admitted as a continuous variable. A linear mixed model was used to assess the changes in diameter and wall thickness across the various time points to examine the time course in arterial adaptations to physical inactivity. In addition, the linear mixed model was also used to assess changes between arteries to examine the local (i.e. common femoral artery) versus systemic (i.e. carotid artery) effects of physical inactivity. All data are expressed as means (SD) and differences were considered to be statistically significant at a *P* value of <0.05.

## Results

### SCI: weeks 3–6

#### Carotid artery

**Fig. 1:**
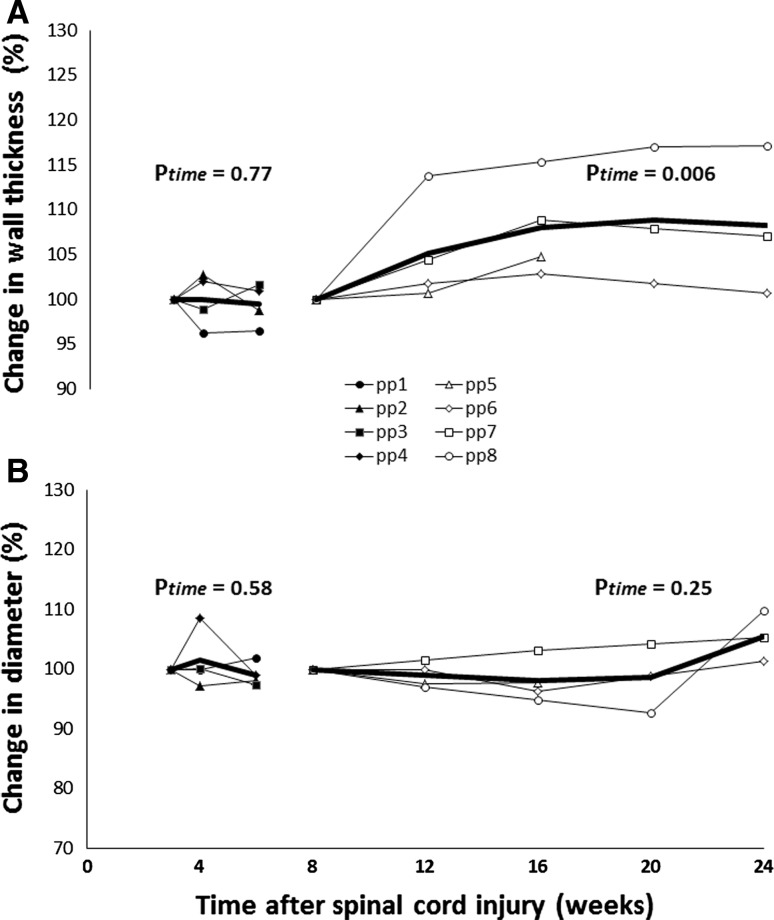
Individual (*thin lines*) and averaged (*thick line*) carotid arterial wall thickness (**a**) and diameter (**b**) from SCI individuals from weeks 3–6 (*n* = 4, *solid symbols*) and weeks 6–24 (*n* = 4, *open symbols*) after a spinal cord injury. Data are presented as the relative change from week 3 or week 8 (set at 100 %). Average data is presented when ≥3 subjects were tested. *P* values from the linear mixed models (based on absolute values) are added for the effect of time

Subject characteristics are presented in Table 1. We found no significant change in carotid artery wall thickness, diameter and wall:lumen ratio (Fig. 1) during weeks 3–6. Also, no change was observed in carotid artery blood flow or shear rate during weeks 3–6 after the injury (Table 2).

**Table 2:** Carotid and common femoral arterial wall thickness, diameter, blood flow and shear rate from SCI individuals during weeks 3–6 (*n* = 4) and weeks 8–24 (*n* = 4) after a spinal cord injury

	Spinal cord injury (weeks 3–6)				Spinal cord injury (weeks 8–24)					
	3 (*n* = 4)	4 (*n* = 4)	6 (*n* = 4)	*P* value	8 (*n* = 4)	12 (*n* = 4)	16 (*n* = 4)	20 (*n* = 3)	24 (*n* = 3)	*P* value
Carotid artery										
Wall thickness (μm)	526 ± 129	526 ± 122	525 ± 135	0.77	471 ± 32	494 ± 13	508 ± 17	508 ± 15	505 ± 13	0.006
Diameter (mm)	6.0 ± 0.3	6.1 ± 0.5	6.0 ± 0.4	0.58	5.6 ± 0.5	5.5 ± 0.4	5.5 ± 0.4	5.4 ± 0.3	5.8 ± 0.8	0.25
Blood flow (ml/min)	333 ± 102	326 ± 025	370 ± 052	0.26	282 ± 63	262 ± 37	249 ± 74	204 ± 65	264 ± 45	0.18
Shear rate (s)	83 ± 18	87 ± 15	102 ± 20	0.15	101 ± 12	106 ± 28	95 ± 38	75 ± 31	97 ± 28	0.23
Common femoral artery										
Wall thickness (μm)	526 ± 169	532 ± 178	540 ± 167	0.10	479 ± 14	509 ± 8	515 ± 18	540 ± 25	557 ± 6	<0.001
Diameter (mm)	7.2 ± 1.2	6.8 ± 1.3	7.0 ± 1.4	0.33	5.7 ± 0.6	6.1 ± 0.7	6.0 ± 0.7	5.8 ± 0.4	6.2 ± 0.1	0.23
Blood flow (ml/min)	249 ± 84	235 ± 123	349 ± 109	0.06	356 ± 160	310 ± 128	279 ± 81	229 ± 80	299 ± 48	0.92
Shear rate (s)	53 ± 13	48 ± 16	71 ± 21	0.023	91 ± 27	87 ± 10	67 ± 12	82 ± 12	56 ± 13	0.63

Data are presented as mean ± SD when three or four subjects were measured at the specific time point. *P* value refers to the linear mixed model (LMM) used to examine the change across time

#### Common femoral artery

**Fig. 2:**
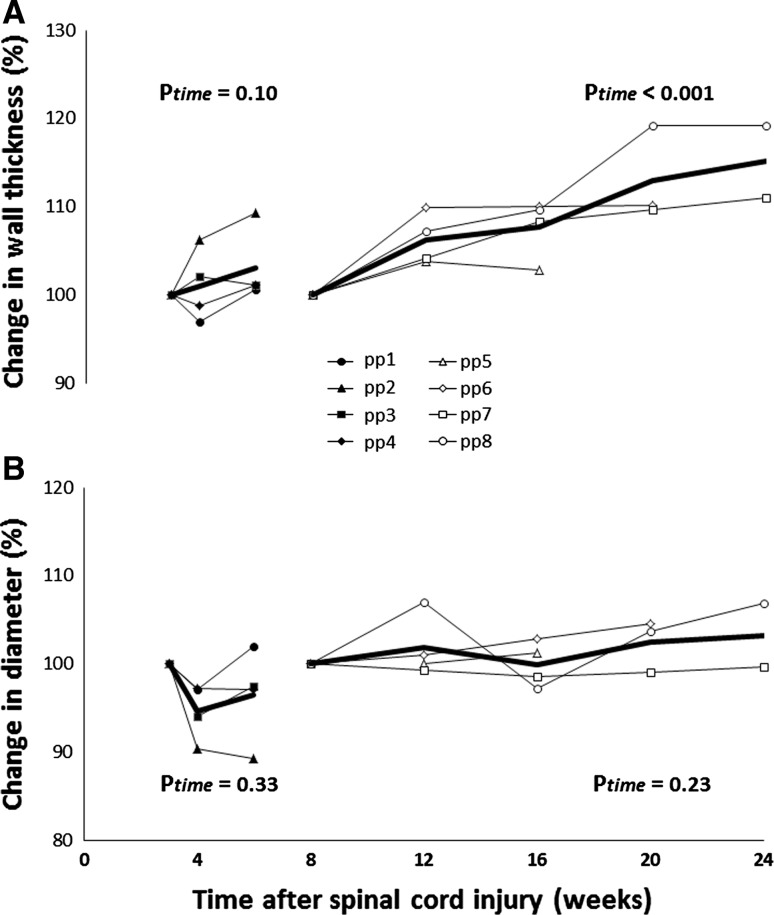
Individual (*thin lines*) and averaged (*thick line*) femoral arterial wall thickness (**a**) and diameter (**b**) from SCI individuals from weeks 3–6 (*n* = 4, *solid symbols*) and weeks 6–24 (*n* = 4, *open symbols*) after a spinal cord injury. Data are presented as the relative change from week 3 or week 8 (set at 100 %). Average data is presented when ≥3 subjects were tested. *P* values from the linear mixed models (based on absolute values) are added for the effect of time

Common femoral artery wall thickness and diameter demonstrated no change between 3 and 6 weeks after the SCI (Fig. 2; Table 2). The change in diameter was accompanied by a significant increase in shear rate over time (Table 2). Baseline blood flow in the femoral artery did not change over time (Table 2).

### SCI: weeks 8–24

#### Carotid artery

We found a gradual, significant increase in carotid artery wall thickness, but no change in carotid artery diameter between 8 and 24 weeks after the SCI (Fig. 1). Carotid artery blood flow or shear rate did not change across this time frame (Table 2).

#### Common femoral artery

Arterial wall thickness demonstrated a gradual, significant increase after 8–24 weeks of the SCI, whilst no change in arterial diameter was observed (Fig. 2; Table 2). Also, no change in blood flow or shear rate was observed in the femoral artery (Table 2).

## Discussion

During the first 6 weeks following SCI, we observed no change in carotid or femoral arterial wall thickness. This was followed by a gradual increase of similar magnitude in both carotid and femoral artery wall thickness between weeks 8 and 24. In contrast, a rapid and localised decrease in diameter, which was completed within 4 weeks after the injury, was evident in the femoral artery feeding the denervated and physically inactive lower limbs (de Groot et al. 2006). These data suggest that conduit arterial wall thickness and diameter do not follow similar patterns of remodelling. The rapid and localised nature of the diameter change, coupled with the lack of further change following prolonged inactivity, suggests that the initial inward remodelling of the femoral artery is associated with the local effects of denervation, rather than the effects of whole body physical activity per se. In contrast, the gradual increase in wall thickness across 24 weeks following SCI and its presence in both the femoral and carotid arteries suggests a systemic impact of whole body physical inactivity. Taken together, our data suggest that the stimuli that underlie adaptation in wall thickness may be independent of those that initiate remodelling of the diameter.

Our analysis of the femoral artery diameter between 3 and 6 weeks after the injury revealed no change, most likely because the presence of a rapid decrease in femoral artery diameter during the first 3 weeks after injury (de Groot et al. 2006). This confirms that local denervation of the lower limbs represents a potent stimulus leading to localised inward remodelling (de Groot et al. 2006; De Groot et al. 2003). In a previous study, we reported that the change in femoral arterial lumen is accompanied by the change in lower limb mass, i.e. an indirect measure of muscle atrophy (de Groot et al. 2006). This suggests that muscle and vascular atrophy in the denervated area after an SCI are related, especially since muscular atrophy also demonstrates strong localised nature. Possibly, the rapid inward remodelling of the artery diameter relates to local changes in shear rate, i.e. a stimulus that importantly contributes to localised arterial remodelling (Langille and O’Donnell 1986; Tinken et al. 2010; Tuttle et al. 2001). In a recent study, we observed larger resting brachial arterial diameter in the preferred limb, compared to non-preferred limb of squash players (Rowley et al. 2011). These differences were not evident in the matched control subjects, suggesting a localized impact on conduit arterial enlargement. Therefore, the localised change in femoral artery diameter may relate to denervation of the lower limbs and the consequent localised change in activity.

The potency of physical inactivity to induce remodelling of arterial wall thickness has been reported before (Matos-Souza et al. 2009; van Duijnhoven et al. 2010). However, our approach allowed for the novel observation that the change in arterial wall thickness follows a different pattern than the change in diameter of the same artery during whole body physical inactivity. Indeed, a gradual increase in femoral arterial wall thickness after an SCI was found, which confirms the widely adopted view that changes in wall thickness are driven by mechanisms that take several months to develop. The larger wall thickness in SCI is in agreement with a previous study that reported a higher carotid artery wall thickness in chronic SCI compared with able-bodied controls (Matos-Souza et al. 2009). Another important novel observation is that the gradual increase in arterial wall thickness was similarly present below (i.e. femoral artery) and above (i.e. carotid artery) the spinal cord lesion, suggesting that remodelling of the arterial wall thickness is driven by systemic, rather than localised, processes. To support this notion, 8-week bed rest leads to a similar increases in wall thickness of the femoral and carotid artery, whilst whole body (resistive) vibration exercise can (partly) prevent this increase in wall thickness (van Duijnhoven et al. 2010). In addition, a recent within (dominant vs. non-dominant) and between subject (squash players vs. controls) comparison showed systemic, rather than localised, changes in conduit arterial wall thickness (Rowley et al. 2011). Collectively, this indicates that whole body physical inactivity is a potent stimulus for systemic changes in arterial wall thickness that occur gradually across time.

Based on the comparable, systemic increase in wall thickness above and below the spinal cord lesion, it is unlikely that local changes in hemodynamic factors, such as shear rate, importantly contribute to the gradual increase in wall thickness. Indeed, we recently found a similar decrease in brachial artery wall thickness after bilateral handgrip exercise training, despite a unilateral attenuated increase in exercise-induced increase in shear rate (Thijssen et al. 2011b). Alternatively, changes in vascular tone may contribute to changes in arterial wall thickness. A higher vascular tone is reported after an SCI, which is mediated through endothelin-1 (Thijssen et al. 2007) and angiotensin-II (Groothuis et al. 2010). The increased vascular tone, but also the pro-atherogenic properties of both vasoconstrictors, (Laughlin et al. 2008) may contribute to the systemic increase in arterial wall thickness. Future studies should further examine mechanisms that contribute to the systemic remodelling of the arterial wall in SCI.

### Limitations

An obvious limitation relates to the small sample size in our study. However, the information presented in this study provides unique insight into the time course of adaptations of the wall thickness immediately after an SCI within subjects. Moreover, wall thickness in carotid and femoral arteries in all subjects demonstrated a consistent and gradual increase after the SCI. Finally, due to the relatively small variation within subjects, our statistical approach demonstrated significant changes across time. Therefore, we believe that our data are valid and may be followed by new studies that will further examine the adaptations of the arterial wall to physical inactivity, and also elucidate its time course of the mechanisms underlying the remodelling.

Another limitation relates to the inclusion of two subjects who smoked until the time of accident. Although evidence indicates that cigarette smoking is related to an increased wall thickness (Bhuiyan et al. 2006), no previous study examined the immediate effects of smoking cessation (as a result of smoking withdrawal during hospitalisation) on arterial wall thickness. In our study, however, we found no change in wall thickness in our subjects during the first 6 weeks after the SCI, whilst a subsequent increase in wall thickness was observed during weeks 6–20. Therefore, we believe that the inclusion of two smokers did not importantly change the major outcomes of our study.

In conclusion, we found a gradual, systemic increase in carotid and femoral artery wall thickness after an SCI, which was preceded by a rapid, localised inward remodelling of the femoral artery that was completed within 4 weeks after the onset of the spinal cord lesion. Accordingly, conduit artery remodelling of the arterial wall and diameter follow a different pattern. These data suggest that stimuli that underlie the systemic and gradual changes in conduit artery wall thickness are different from those factors that initiate the local, rapid inward remodelling of the diameter during physical inactivity in humans.
